# Targeted capture to assess neutral genomic variation in the narrow-leaf hopbush across a continental biodiversity refugium

**DOI:** 10.1038/srep41367

**Published:** 2017-02-01

**Authors:** Matthew J. Christmas, Ed Biffin, Martin F. Breed, Andrew J. Lowe

**Affiliations:** 1 Environment Institute and School of Biological Sciences, The University of Adelaide, North Terrace, SA 5005, Australia; 2 State Herbarium of South Australia, Hackney Road, Adelaide, SA 5000, Australia

## Abstract

The Adelaide geosyncline, a mountainous region in central southern Australia, is purported to be an important continental refugium for Mediterranean and semi-arid Australian biota, yet few population genetic studies have been conducted to test this theory. Here, we focus on a plant species distributed widely throughout the region, the narrow-leaf hopbush, *Dodonaea viscosa* ssp. *angustissima*, and examine its genetic diversity and population structure. We used a hybrid-capture target enrichment technique to selectively sequence over 700 genes from 89 individuals across 17 sampling locations. We compared 815 single nucleotide polymorphisms among individuals and populations to investigate population genetic structure. Three distinct genetic clusters were identified; a Flinders/Gammon ranges cluster, an Eastern cluster, and a Kangaroo Island cluster. Higher genetic diversity was identified in the Flinders/Gammon Ranges cluster, indicating that this area is likely to have acted as a refugium during past climate oscillations. We discuss these findings and consider the historical range dynamics of these populations. We also provide methodological considerations for population genomics studies that aim to use novel genomic approaches (such as target capture methods) on non-model systems. The application of our findings to restoration of this species across the region are also considered.

Ecological and evolutionary responses to contemporary climate change are evident in many species around the world^1^,^2^. The impacts of a changing climate are predicted to continue to have widespread effects as conditions become more extreme^3^,^4^. Persistence of plant populations under climate change will be in large part driven by their ability to overcome constraints to migration and adaptation^1^,^5^,^6^. For example, large populations with high genetic diversity and connectivity to neighbouring populations should be able to maximise adaptive responses to environmental change whereas small, inbred populations may lack the genetic diversity for selection to act on.

Even if populations are able to maintain high genetic diversity, connectivity and dispersal, rapid and/or extreme climate change can push species beyond their adaptive limits in at least parts of their range. During past climate oscillations, particularly those experienced during the Pleistocene, refugia are thought to have played a major role in the persistence of a vast number of species^7^,^8^,^9^. Refugia are areas that provide species with spatial and/or temporal protection from disturbances^10^ and, under climate change, can act as safe havens and shelter species from the harshest conditions. For example, the Adelaide geosyncline region in South Australia (Fig. 1), the focal region of this study, has been identified as an important historical refugium. This ancient rift complex extends over 800 km, from Kangaroo Island in the south to the most northern extent of the Flinders Ranges. It encompasses two mountain ranges: the Mount Lofty and Flinders Ranges, with a highest peak of 1,189 m (St Mary Peak). In particular, Kangaroo Island and the Flinders Ranges are thought to have acted as refugia for species to retreat to during colder drier periods^7^,^11^.

Under contemporary climate change, the Adelaide geosyncline has the potential to offer refuge from climate extremes, providing altitudinal and latitudinal gradients for species to migrate across. However, the capacity of this area to be an effective future refugium may be compromised by its highly fragmented state, where habitat modification over the last 200 years has led to little of the historical woodlands and forest remaining^12^,^13^,^14^. Despite its potential importance, the area remains largely understudied in terms of the population genetic structure and diversity of component species. To our knowledge, only one other published plant population genetic study has focussed on this region^15^.

Contractions to and expansions from refugia leave genetic signatures across the genome, which contribute to the structuring of genetic diversity in contemporary populations. Populations persisting in past refugia generally maintain higher genetic diversity than the populations that have expanded from them^16^,^17^. Measures of genetic diversity and structure in contemporary populations therefore allow us to make inferences about past responses to climate change.

In this study, we focused on the narrow-leaf hopbush, *Dodonaea viscosa* ssp. *angustissima (D. v. angustissima* hereafter), a widely distributed endemic woody shrub of Australia with a range extending throughout the southern and central regions of the continent. Its hardy nature is reflected in its wide distribution across diverse habitats such as open woodlands, sand plains, and on margins of sand dunes^18^. We sampled *D. v. angustissima*’s distribution across the Adelaide geosyncline region (Fig. 1). This region spans a wide temperature and rainfall gradient, with cooler, wetter conditions in the south and warmer, drier conditions in the north and east.

The southern extent of the study region has been largely cleared since European settlement with, for example, less than 10% of the original vegetation cover remaining in the Mount Lofty Ranges^12^,^13^,^14^. *D. v. angustissima* is commonly used in restoration projects throughout this region, however very little is known about the level and structure of genetic diversity and there is an increasing call for this type of information to be incorporated into restoration planning^19^,^20^,^21^. In particular, measures of population genetic diversity and structure can help ensure the sourcing of high quality and genetically diverse seed in order to maximise adaptive potential of restored populations under climate change^21^.

With the onset of the ‘genomics era’, genome-wide data are now straightforward to generate for non-model species^22^. Genome-wide datasets are superior to more traditional genetic markers (e.g. microsatellites) in estimating the levels and structuring of population genetic diversity^23^,^24^. For example, the use of hundreds to tens of thousands of single nucleotide polymorphism (SNP) markers distributed throughout the genome means that population genetic studies no longer need as many individual samples per population for accurate allele frequency estimates as was needed when measuring relatively few microsatellite markers^25^,^26^. As a result, more populations can be included in a study without added expense.

We utilised a novel target capture method to identify single nucleotide polymorphisms (SNPs) present across our samples. We genotyped 89 *D. v. angustissima* samples from 17 populations to examine population genetic structure and diversity across the understudied Adelaide geosyncline. Genetic structure analyses were performed to assess population connectivity. Measures of genetic diversity were calculated within and among populations and identified genetic clusters in order to assess the distribution of genetic diversity across this region. We used these measures to determine the level of support for the hypothesis that populations within the Flinders ranges are remnants of a past refugium. This may be indicated by distinct genetic clustering and elevated levels of genetic diversity within the Flinders Ranges, as has been observed in previous population genetic studies across the region^15^,^27^.

## Results

### Sequence data, SNP filtering and outlier analysis

Sequencing of hybrid-capture libraries from all 89 individuals resulted in a total of ~332 million reads, with the number of reads sequenced per individual ranging from 2.3 million to 5 million (mean 3.6 million reads per individual). The percentage of reads that mapped back to the transcriptome reference was 15.7%, which is low but not to be unexpected with the approach taken. Targeted sequencing using hybrid-capture baits is a relatively new approach, particularly for organisms without reference genomes. A similar approach was used in a study of grey wolf genomic variation and they achieved mapping success of ~86% of raw reads mapping to the dog reference genome^28^. A mapping success of ~33% was achieved in a study of genomic variation in *Heliconius* butterfies using a targeted sequencing approach^29^. In both of these studies, capture design and mapping were performed using genomic rather than transcriptomic sequences. By designing capture baits based on a transcriptome reference, alternate splicing and introns, for example, cannot be accounted for, which results in the sequencing of genomic regions that will not map to the transcriptome. This explains our low mapping success compared to other studies.

Of the reads that mapped, 67.7% mapped in pairs. Following the calling of variants by identifying SNP differences between the reference and mapped sequences, rigorous and stringent filtering steps were taken to provide a reliable set of neutral SNP calls with high coverage across all individuals. Filtering of raw SNPs on depth of coverage, minimum minor allele frequency, and percentage of missing data per SNP resulted in a set of 25,329 SNPs. These SNPs were then pruned of SNPs in LD, reducing the SNP set to 8,462. The requirement of at least 100 bp between each SNP reduced the SNP set further to 2,800 SNPs. We excluded an additional 342 *F*_*ST*_ outlier SNPs as they were deemed to be non-neutral. Of the remaining 2,458 SNPs, a further 1,643 SNPs were removed for having negative *F*_*IS*_ values as a conservative method of excluding potential paralogous SNPs. This resulted in a final SNP set of 815 SNPs for population genetic diversity and structure analysis.

### Population genetic structure

**Figure 1:**
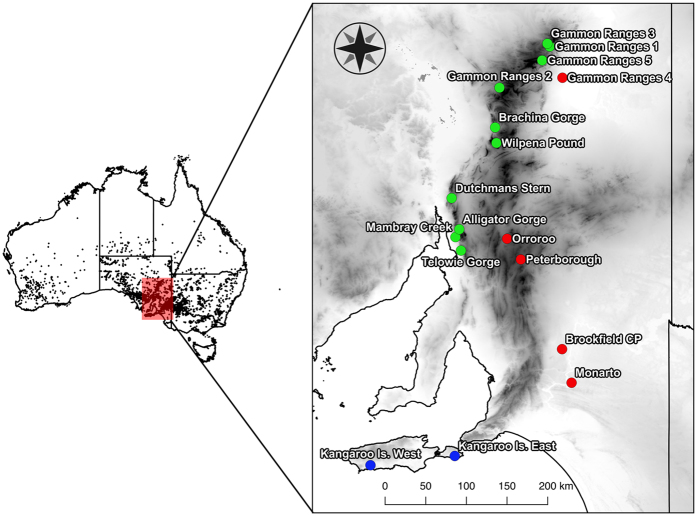
Map of sampling region in South Australia. Population sampling locations of *Dodonaea viscosa* ssp. *angustissima* are indicated by coloured circles, where colours represent genetic cluster assignment from population genetic structure analysis: blue = Kangaroo Island cluster; green = Flinders/Gammon ranges cluster; red = Eastern cluster. Map shading represents elevation with darker shading indicating higher elevation (© Commonwealth of Australia (Geoscience Australia) 2016). Black dots on Australian continent map represent all post-1980 *D. viscosa* ssp. *angustissima* sampling locations, downloaded from the Atlas of Living Australia (Atlas of Living Australia occurrence download at http://www.ala.org.au. Accessed 3 June 2016). The figure was generated using Quantum GIS Geographic Information System (Quantum GIS Development Team, 2016, Open source geospatial foundation project, http://qgis.osgeo.org).

**Figure 2:**
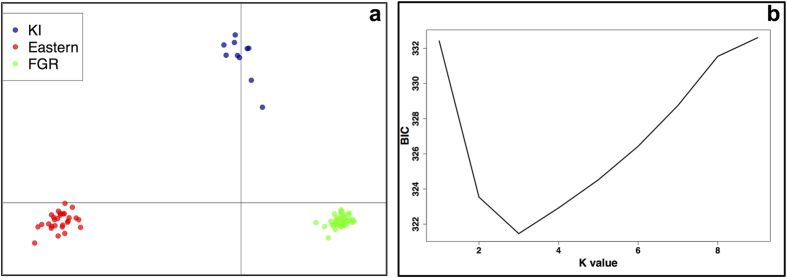
Discriminant analysis of principal components (DAPC) results. (**a**) Principal component scatter plot of all individuals, based on the DAPC output, and (**b**) the optimal number of clusters (*K*) as determined by ‘k-means’, a clustering algorithm which looks for the value of *K* that maximises the variation between groups. The Bayesian Information Criterion (BIC) is plotted for *K* = 1–9 and the ‘elbow’ in the graph at *K* = 3 indicates this to be the most likely value of *K*. KI = Kangaroo Island cluster, Eastern = Eastern cluster, FGR = Finders/Gammon Ranges cluster.

**Figure 3:**
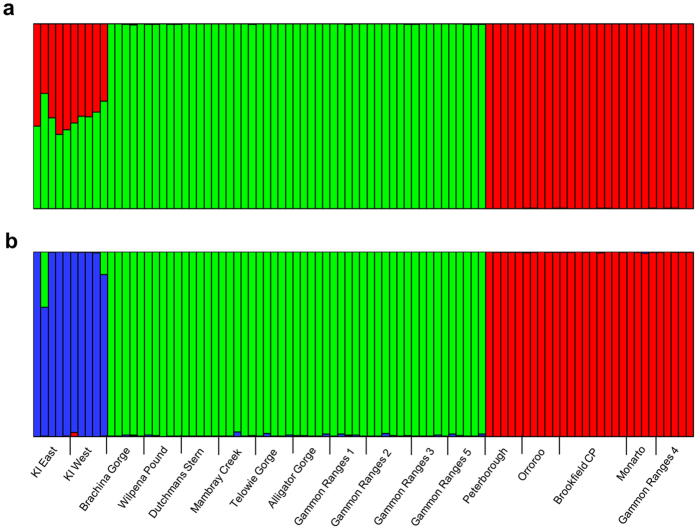
Individual genetic cluster assignments from STRUCTURE results. Results shown are the combined results from ten replicate runs per K value using the admixture model with 200,000 burn in followed by 1,000,000 iterations. (**a**) *K* = 2 (most likely, ΔK = 9,835.32) and (**b**) *K* = 3 (second most likely, ΔK = 1,716.64). Coloured bars represent percentage assignment of individuals to each of the two (**a**) or three (**b**) identified clusters. Sampling site locations are listed across the bottom.

In a discriminant analysis of principal components (DAPC), *K* = 3 had the lowest Bayesian information criterion (BIC) value, with a clear ‘elbow’ in the graph at this *K* value (Fig. 2). Two discriminant functions were retained, explaining 84.1% of the variance. Three distinct clusters were identified, one containing all Kangaroo Island samples (KI cluster), one containing samples from within the Flinders and Gammon Ranges (FGR cluster), and one containing all samples to the east of the ranges (Eastern cluster) (Figs 1 and 2). The STRUCTURE analysis revealed two to be the most likely value of *K* (ΔK = 9,835.32) with *K* = 3 the second most likely (ΔK = 1,716.64). When *K* = 2, the same FGR cluster and Eastern cluster as in the DAPC analysis were identified, with the Kangaroo Island samples having ~50% assignment to each of these clusters (Fig. 3a). When *K* = 3, the Kangaroo Island samples formed a third distinct genetic cluster (Fig. 3b), matching the DAPC results.

Nested AMOVA analysis revealed that the majority of the genetic variance was within individuals (69%, Table 1). There was very little variation among individuals within sample populations or among populations within the identified genetic clusters (Table 1). Among genetic cluster variance was significant and equal to 16.9% of the total variance (Table 1), supporting the clustering identified by the DAPC and STRUCTURE analyses. Average pairwise *F*_*ST*_ estimates indicated the greatest differentiation was between the Eastern and KI clusters (*F*_*ST*_ = 0.280), with the least differentiation between the FGR and KI clusters (*F*_*ST*_ = 0.138). Genetic differentiation between the FGR and Eastern clusters was intermediate to these values (*F*_*ST*_ = 0.189).

### Genetic diversity

Overall observed (H_O_) and expected (H_E_) heterozygosity were 0.123 (95% CI = ±0.007) and 0.141 (95% CI = ±0.007) respectively, with lowest H_O_ and H_E_ in the Peterborough subpopulation, greatest H_O_ in the Telowie Gorge population and greatest H_E_ in the Brachina Gorge population (Table 2). The FGR cluster had the highest genetic diversity, with the Eastern and KI clusters harbouring similarly lower levels (Table 2).

### Isolation by distance

**Figure 4:**
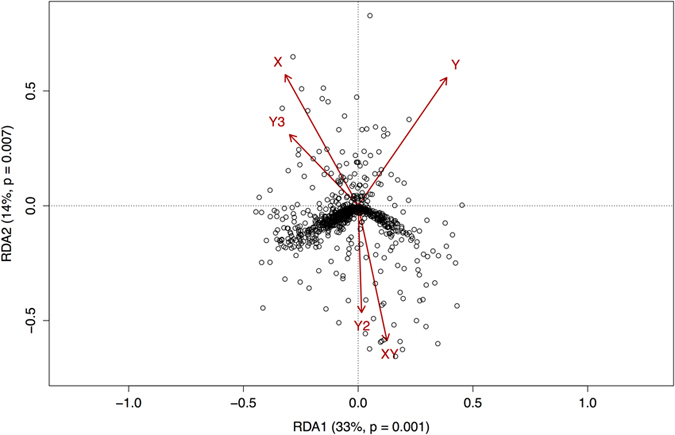
Redundancy analysis (RDA) biplot representing the output of an RDA performed on allele frequency data from 89 *Dodonaea viscosa* ssp. *angustissima* samples from 17 populations. Open circles represent the ordinated allele frequencies (response variable); Red arrows represent spatial polynomials (explanatory variables) plotted as vectors. 58% of the total variation in the genetic data was constrained by the spatial explanatory variables. Of this constrained variation, 33% (p = 0.001) was constrained by axis one (RDA1), and 14% (p = 0.007) by axis two (RDA2). Significance of RDA was assessed using an analysis of variance (ANOVA).

Redundancy analysis (RDA) performed on all samples demonstrated that 58% of the total genetic variation was constrained by spatial variables (ANOVA, F = 3.078, P < 0.001; Fig. 4). By multiplying the percentage of constrained variation (58%) by the overall *F*_*ST*_ (0.153) we ascertained that the proportion of the total genetic variation that is explained by the spatial variables is equivalent to an *F*_*ST*_ of 0.089. For the Flinders/Gammon Ranges cluster, 15.8% of the total genetic variation was constrained by latitude (ANOVA, F = 1.50, P < 0.01). Overall *F*_*ST*_ in this cluster was 0.044, and so the proportion of the total genetic variation explained by the spatial variables is equivalent to an *F*_*ST*_ of 0.007. Spatial variables did not explain significant levels of total genetic variation in the Eastern cluster.

## Discussion

Our analysis of neutral SNP variation, distributed across 411 genes, in *D. v. angustissima* detected strong signals of population genetic structure throughout the Adelaide geosyncline region, identifying three distinct clusters. Populations sampled along the Flinders and Gammon Ranges, a significant mountain range in the region, showed distinct genetic signals from populations sampled to the east of the ranges, as well as those from Kangaroo Island. The Flinders and Gammon Ranges cluster demonstrated higher genetic diversity across the sequenced genes compared to the other two clusters. This provides evidence towards the hypothesis that the Flinders Ranges has acted as a refugium for *D. v. angustissima* in the past, as has been suggested for several other species^15^,^27^.

The presence of three distinct genetic clusters identified among our sampled populations suggests that gene flow among these locations is low, which is perhaps surprising. There is evidence of *D. viscosa* pollen reaching Macquarie Island in the southwest Pacific Ocean^30^, ~1,500 km from Tasmania, demonstrating the species’ capacity for pollen dispersal over very large distances. Therefore, pollen dispersal over the much shorter distances between the populations we have sampled here is likely. We therefore consider different explanations as to why these distinct genetic clusters exist.

### A past refugium?

The Flinders Ranges, with its varied topography and high elevation, provides ideal refugial conditions enabling species to remain within their preferred climatic envelopes with only short migration distances^10^. The region has been identified as a refugium for the needle bottlebrush (*Melaleuca orophila*) during Mid-Pleistocene climate oscillations^15^. The high genetic diversity found within the populations we sampled within the Flinders Ranges, compared to surrounding populations, suggests it may have played a similar role for *D. v. angustissima*. Evidence for the presence of *D. v. angustissima* in the Central Flinders Ranges (specifically Brachina Gorge) during the Early to Mid-Holocene has been found in an analysis of stick-nest rat middens^31^. Whilst this does not go as far back as the Pleistocene, it does suggest that the species has been prevalent in the area for an extended time.

### East-west divide

A steep rainfall gradient exists across the Flinders Ranges, with rainfall rapidly decreasing to the east of the ranges. This means that populations in the FGR and Eastern clusters are inhabiting contrasting environments, in terms of rainfall at least. For example, Gammon Ranges population four was less than 35 km from population five, yet the two populations fall into distinct genetic clusters. Considering the high genetic similarity between all FGR populations, which extend over a much larger distance, gene flow would be expected between these two populations. The two sampling sites differ greatly in their elevation (27 m at GR4 versus 700 m at GR5), their annual mean precipitation (13 mm at GR4 and 24 mm at GR5), and their annual mean aridity index values (0.07 at GR4 and 0.14 at GR5), so gene flow may be unsuccessful despite the short distance, resulting in isolation by environment (precipitation and aridity data obtained from the Atlas of Living Australia, June 2016).

An alternative, or perhaps complementary explanation for the observed genetic differentiation between the FGR and Eastern clusters is that the region may represent a contact zone between distinct range expansions, with the eastern samples representing the edge of a range expansion from the southeast. A lack of admixture between these two genetic sources would result in the patterns we observe here. This is also supported by the lower differentiation between the FGR and KI clusters compared to that between the FGR and Eastern clusters. Further sampling of more eastern populations would help to ascertain the origin of the Eastern cluster.

### Kangaroo Island differentiation

The STRUCTURE analysis provided most support for two distinct clusters, with the KI samples being an admixture of the FGR and Eastern clusters. Admixing of the FGR and Eastern clusters suggests that the KI populations may have experienced gene flow from the mainland. KI is only 13.5 km offshore; *D. v. angustissima* seed can remain viable in sea water for extended periods of time^32^ and it is hypothesised that the species has dispersed out of Australia to as far as South America and Madagascar^33^. Coupled with the evidence for long distance pollen dispersal in this species discussed earlier, gene flow from the mainland to KI is a real possiblility.

In the DAPC analysis, the KI populations were identified as genetically distinct from the mainland populations, despite the possibility for long distance gene flow. Differentiation between the mainland and KI populations may be explained by prolonged separation of these populations. The contemporary ranges of mainland populations of *D. v. angustissima* do not extend to coastal regions of the Fleurieu peninsula, the closest part of the mainland to KI. Also, KI has been separated from the mainland since the retreating ice sheets led to sea level rise at the end of the Pleistocene, around 10,000 years ago^34^. There is also the possibility that the KI populations are more closely related to unsampled populations from the Yorke and/or Eyre Peninsulas, west of Adelaide. Further sampling would need to be undertaken to test this.

### Isolation by distance is not the answer

Redundancy analysis showed that 58% of the genetic variation across all samples could be explained by spatial location of populations, suggesting isolation by distance. However, as most of the genetic variation was distributed among genetic clusters as well as the fact that the three identified clusters are (mostly) spatially separated, the constrained variation cannot be attributed solely to isolation by distance. Testing for the influence of space on within-cluster variation found that spatial location explained only a small percentage of genetic variation in the FGR cluster and did not significantly explain any in the Eastern Cluster. This adds to the evidence that most of the genetic variation is distributed among the identified clusters, rather than within.

### Developing genomic resources for non-model species

The target capture method used in the current study^35^,^36^ is yet to be widely utilised in the fields of population and conservation genetics, in comparison to other genome partitioning methods such as Genotyping by Sequencing (GBS) and RADSeq^37^. Here, we chose a more targeted approach as it allowed us to sequence specific genes of interest identified and designed from the assembled transcriptome for the species^38^. This resulted in reliably sequencing over 700 gene regions with putative functions assigned for each individual. The main advantage of this approach was that, for a non-model organism without a reference genome, identified variants could be assigned to the specific gene they occurred in and their functional significance could be ascertained. Although this type of information is not necessarily informative for population genetic analyses, where the aim is to estimate neutral processes, the development of such a genetic marker dataset provided the neutral markers required for the types of analyses presented here (as most of the variation, even in functional genes, is expected to be neutral) as well as providing a set of markers located within transcribed genes that can be explored for evidence of non-neutral processes such as selection^39^.

### Sampling design

In our study, 5–8 individuals were sampled per population. These relatively small numbers were constrained by the fact that, as in most population genetic studies, compromises must be made between the number of populations sampled and the number of samples per population due to budget restrictions. This trade-off between per-population sample size and number of populations when using NGS genotyping methods has led to several published studies having fewer than ten individuals per population^40^,^41^. This is a potential issue as estimates of *F*_*ST*_ can be biased if sample sizes are too small^42^,^43^,^44^. It has been suggested that power in *F*_*ST*_ estimates can more readily be increased by sampling more individuals per population rather than sampling more markers per individual, particularly when *F*_*ST*_ is low^44^. However, with the advent of next-generation sequencing, it is now cheaper to increase the number of markers compared to increasing the number of individuals genotyped. In their simulations of the effect of number of individuals on inferential power for different number of SNPs, Morin *et al*.^44^ demonstrated that a sample size of 10 individuals per population and only 20 SNPs provided complete power to detect differentiation at the level of *F*_*ST*_ = 0.2. As few as four samples per population have been shown to be sufficient for *F*_*ST*_ estimates when using a large number of markers (>1,000)^25^,^26^.

Average pairwise *F*_*ST*_ among our sampled populations was 0.16, which is quite low, and so our use of only 5–8 individuals per population may have resulted in low power for our *F*_*ST*_ estimates. However, the assignment of individuals to genetic clusters through the genetic structure analyses meant that we were actually working with sample sizes of 51, 28, and 10 for the FGR, Eastern and KI clusters respectively. This, along with our use of a large number of SNPs (815) should have provided sufficient power to reliably detect differentiation among the clusters without having to compromise on the number of sampling sites.

### Conservation and restoration implications

The Adelaide Geosyncline has a number of National and Conservation Parks where natural stands of native vegetation are protected. Between these protected areas much land has been cleared, leaving protected areas fragmented across the landscape. Large-scale restoration is carried out across the region to increase the cover of native vegetation, re-connect these fragments, and return functional, native ecosystems. Recent work has focussed on improving success rates of plantings under climate change, due to the questionable success rates of locally sourced material^19^,^20^,^21^. Supplementing local gene pools to increase their adaptive potential should provide restored populations with better chances of thriving into the future, whilst avoiding outbreeding depression and maladaptation to local conditions^19^,^20^. For *D. v. angustissima*, a species commonly used in revegetation projects, the distinct genetic clustering and clear assignment of individuals to these clusters demonstrates that the three populations are genetically isolated from one another, and adaptive differences are likely to be present. As such, movement of seed between these regions may result in maladapted plants and outbreeding depression. Further investigation into the phenotypic differences among plants across these genetic clusters through reciprocal transplant experiments are required to fully assess the risks of mixing seed from across the identified genetic clusters.

## Methods

### Study system and sampling

We sampled *D. v. angustissima* throughout the Adelaide geosyncline, with sampling effort stretching from Kangaroo Island in the south, through the Mount Lofty and Flinders Ranges to the Gammon Ranges in the north (Fig. 1). This sampling design enabled us to collect samples covering multiple environmental gradients, with a strong north-south temperature and rainfall gradient as well as an independent east-west rainfall gradient. Avoiding a single, latitudinal transect for sampling and sampling populations that are geographically close but environmentally dissimilar makes the detection of population genetic structure driven by adaptation (isolation by ecology) as well as by distance possible, as large environmental distances between populations could lead to genetic differentiation resulting from local adaptation^45^,^46^. *D. v. angustissima* leaf samples were collected from 89 plants, which included 5–8 plants per site at 17 sites across the region. Leaf samples were stored in teabags on silica gel prior to DNA extraction.

### Genome-wide data generation

#### Capture probe design

The previously published transcriptome for this species^38^ was used to design hybrid-capture probes for selectively sequencing hundreds of gene regions reliably across all samples. Previous annotation of the transcriptome via BLAST searches to the NCBI non-redundant database meant that genes and their putative functions had already been identified (details in ref. 38). This information was used to design a probe set that could generate data on functional regions of the genome to inform on both neutral (the present study) and adaptive (a separate study^39^) genetic variation. Functional information was used to select a set of 353 genes that were assigned gene ontology classifications relating to a response to water stress as well as, more specifically, all genes identified as relating to aquaporin and abscisic acid (ABA) functions. A second set of 617 genes was also selected on the basis of the presence of non-synonymous SNPs in a subspecies comparison in ref. 38. This resulted in a set of 970 target genes. Hybrid capture probes for the capture of these 970 genes were designed and synthesised by MYcroarray (MI, USA) using their 80-mer MyBaits custom bait library system with 2x tiling. RepeatMasker (http://www.repeatmasker.org) was used to mask interspersed repeats and low complexity DNA sequences based on the *Arabidopsis thaliana* genome during the bait design.

Although the targeted gene sequences were mainly selected based on *a priori* expectations that they may be under selection and so informative for a separate study focussing on signatures of selection^39^, it is expected that a significant proportion of the variation in these targeted genes will be neutral. By identifying the neutral variation in this dataset we were able to use it to address questions of neutral population genetic diversity and structure in the current study.

### DNA extraction, hybrid-capture enrichment and sequencing

DNA was extracted using the Machery-Nagel Nucleospin Plant II Kit at the Australian Genome Research Facility (AGRF, Adelaide, Australia). The extracted DNA was then sonicated for random sheering and Illumina’s TruSeq Nano DNA protocol was used for size selection and sequencing adapter and barcode ligation. The hybrid-capture enrichment reactions were carried out following the MyBaits protocol v.2 (www.mycroarray.com/pdf/MYbaits-manual-v2.pdf) using the high stringency wash buffer and 12 cycles of post-capture PCR. Following capture 100 bp paired-end sequencing with dual indexing of 89 samples was performed on one lane of an Illumina HiSeq 2000 at AGRF (Melbourne, Australia). Sequence data was subsequently processed using the Illumina CASAVA pipeline (version 1.8.2).

### Sequence quality, SNP discovery and filtering

Sequence quality was assessed using FastQC (http://www.bioinformatics.babraham.ac.uk/projects/fastqc/). Raw sequence quality was very high, negating the need for any trimming. Mapping of raw sequence reads to the reference transcriptome from^38^ was performed using BWA^47^. The indexed reference was created using default settings. Picard tools (http://broadinstitute.github.io/picard/) were used to compress the resulting SAM files, sort the sequences by reference contig and mark duplicated sequence reads. Mapping characteristics were assessed using SAMtools^48^. Variant calling was performed per individual on the mapped reads using the SAMtools utility “mpileup”. Settings used are listed in the Supplementary Methods. Variants were output as genotype probabilities in one VCF file per individual. Output VCF files were then merged and genotypes were called from the genotype probabilities using the bcftools “call” function with the ‘consensus-caller’ flag.

SNPs were subsequently filtered using VCFTools^49^ as follows: minimum depth of 10 reads per individual, minor allele frequency >10%, missing data per SNP <25% across all individuals. The mean number of base pairs between SNPs for each contig was also calculated and contigs containing fewer than 10 base pairs per SNP were removed in order to control for mapping errors. We then filtered out SNPs that were likely to be in linkage disequilibrium (LD) using the LD pruning tool in PLINK (http://pngu.mgh.harvard.edu/~purcell/plink/). This ran independent pairwise regressions between all SNPs. A cut-off r^2^ > 0.5 was used, whereby one of a pair of SNPs was removed from the dataset if the coefficient of determination between the pair was greater than 0.5, thus removing SNPs showing strong signals of LD. A further requirement of at least 100 bp between each SNP was also implemented.

We then removed outlier SNPs using an *F*_*ST*_-based outlier analysis implemented in BayeScan ver. 2.0^50^. BayeScan implements a reversible-jump MCMC algorithm to estimate the posterior probability of models of neutrality and selection. The use of posterior probabilities adjusts for inflated false discovery rates (FDR; the expected proportion of false positives among outlier markers). Q-values (the minimum FDR at which a locus may become significant) are calculated for each locus and used to set an FDR threshold of 0.05 (a 5% false positive rate). Default settings were used, including prior odds = 10. Such low prior odds increase the risk of false positives^51^ and therefore will result in a very conservative set of neutral SNPs, which in our case is ideal.

The hybrid capture baits were designed based on transcriptome sequences and, as a reference genome was lacking, the presence of duplicated or paralogous sequences of the bait targets within the *D. v. angustissima* genome was unknown. If present, paralogous sequences may map together during the mapping stage. This could skew allele frequency estimates and bias results. Paired-end sequencing was employed in this study, and the requirement of both members of a pair to be present when mapping to a reference can help to reduce the chance of mapping paralogous regions together. As an extra control, *F*_*IS*_ values of the generated SNP set were calculated in GENODIVE and SNPs displaying significantly negative *F*_*IS*_ values (indicating greater than expected heterozygosity under Hardy-Weinburg equilibrium, which may be indicative of paralogous regions mapping together; significance assessed using permutation tests with 10,000 permutations) were removed using VCFTools.

### Population genetic analysis

#### Genetic clustering analysis

Population genetic clustering analyses were performed in order to group genetically-similar individuals together. We used a non-model based method called a discriminant analysis of principle components (DAPC^52^), and the model-based method STRUCTURE^53^. Firstly, DAPC^52^, implemented in adegenet in R^54^, was used in order to ascertain the number and assignment of individuals to genetic clusters. DAPC is a non-model-based multivariate approach, which seeks discriminating functions between groups of individuals while minimising variation within clusters. Genetic data were first transformed into uncorrelated components using principal component analysis (PCA). The number of genetic clusters was then defined using k-means, a clustering algorithm that looks for the value of k that maximises the variation between groups. The Bayesian Information Criterion (BIC) was calculated for *K* = 1–10 and the *K* value with the lowest BIC was selected as the optimal number of clusters. A discriminant analysis was then performed on the first 40 principal components using the function dapc, implemented in R, in order to efficiently describe the genetic clusters and assign samples to each cluster.

Secondly, the most likely number of clusters and individual assignment to those clusters was assessed using STRUCTURE ver. 2.3.4. An admixture model was used to determine the number of population clusters (K) with a burn-in of 200,000 followed by 1,000,000 iterations. *K* values 1–10 were assessed, with 10 replicates per *K* value. ΔK^55^ was calculated for each *K* value in Structure Harvester ver.0.6.94^56^ in order to assess the most likely *K*. Results from replicate runs of the most likely *K* were combined using CLUMPP^57^ with default settings.

#### Analysis of molecular variance

A nested analysis of molecular variance (AMOVA)^58^ was performed to assess within and among population genetic differentiation. This was performed in GENODIVE^59^, with individuals nested within populations and populations nested within the genetic clusters identified by genetic structure analysis. Fixation indices and the proportion of genetic variation found within individuals (*F*_*IT*_), among individuals nested within populations (*F*_*IS*_), among populations nested within genetic clusters (*F*_*SC*_), and among genetic clusters (*F*_*CT*_) were calculated. Significance of each fixation index was evaluated using permutation tests with 10,000 permutations in order to assess the partitioning of genetic variation among subpopulations as well as among the genetic clusters. Pairwise *F*_*ST*_^60^ was calculated between each of the genetic clusters identified by the structure analyses. Genetic diversity was assessed through measures of expected and observed heterozygosity for each sampling site, as well as for the genetic clusters determined by population structure analyses, in GENODIVE ver. 2.0b27^59^.

#### Redundancy analysis

In order to measure the spatial component of the among-population variation a redundancy analysis (RDA) was performed on the population allele frequencies using a modified R script from^46^. Briefly, allele frequencies for one allele per locus were calculated for each population. A matrix of spatial variables was made by calculating orthogonal third-degree polynomials based on population coordinates using the command “poly” in R^46^,^61^. The command “OrdiStep” in the R package VEGAN was used for forward selection of spatial variables in order to prevent overfitting. RDA was then performed, using the command “rda” (VEGAN), with the allele frequency matrix as dependent and spatial polynomials matrix as independent variables. The output from the RDA was then used to calculate the percentage of the total genetic variation that is explained by the spatial variables by multiplying the proportion of constrained variation with the overall value of *F*_*ST*_^46^. ANOVA was used to assess the significance of the RDA.

In order to account for the fact that identified genetic clusters were geographically disparate, the RDA analysis was performed separately on only populations from the FGR cluster, only populations from the Eastern cluster, as well as all samples together. The Kangaroo Island populations were not analysed separately due to the low number of samples and limited geographic variation.

## Additional Information

**Accession codes:** Sequence reads are archived at the NCBI SRA with accession number SRP077342. The variants file is available as a supplementary file in variant call format (Neutral_SNPs_File.vcf).

**How to cite this article**: Christmas, M. J. *et al*. Targeted capture to assess neutral genomic variation in the narrow-leaf hopbush across a continental biodiversity refugium. *Sci. Rep.* **7**, 41367; doi: 10.1038/srep41367 (2017).

**Publisher's note:** Springer Nature remains neutral with regard to jurisdictional claims in published maps and institutional affiliations.

## Supplementary Material

Supplementary Information

Supplementary Dataset 1

## Figures and Tables

**Table 1:** Nested analysis of molecular variance (AMOVA).

Source of Variation	Nested in	%var	F-stat	F-value	P
Within Individual	—	69.3	*F*_IT_	0.31	—
Among Individual	Population	9.7	*F*_IS_	0.12	<0.001
Among Population	Genetic clusters	4.1	*F*_SC_	0.05	<0.001
Among genetic clusters	—	16.9	*F*_CT_	0.17	<0.001

Individuals (*n* = 89) are nested within populations (*n* = 17), and populations are nested within genetic clusters identified from genetic structure analyses (*n* = 3). The significance of the F statistics was tested using 10,000 permutations in a series of permutation tests.

**Table 2:** Observed and expected heterozygosity for the 17 sampling sites.

Sampling site	*n*	H_O_	H_E_
Kangaroo Is. East	5	0.073	0.070
Kangaroo Is. West	5	0.068	0.078
Peterborough	5	0.066	0.076
Orroroo	5	0.074	0.083
Brachina Gorge	5	0.167	0.202
Wilpena Pound	5	0.129	0.167
Dutchmans Stern	5	0.141	0.163
Mambray Creek	5	0.157	0.181
Telowie Gorge	5	0.168	0.181
Alligator Gorge	5	0.150	0.168
Brookfield CP	8	0.086	0.097
Monarto	5	0.075	0.092
Gammon Ranges 1	5	0.163	0.182
Gammon Ranges 2	6	0.163	0.184
Gammon Ranges 3	5	0.165	0.185
Gammon Ranges 4	5	0.080	0.092
Gammon Ranges 5	5	0.165	0.194
**Genetic clusters**			
Kangaroo Is.	10	0.071	0.079
Flinders/Gammon	51	0.157	0.189
Eastern	28	0.077	0.092
**Overall**	89	0.123	0.141

The number of individuals sampled per population (*n*), and observed (H_O_) and expected (H_E_) heterozygosity.
