# Supplementary material for: Targeted capture to assess neutral genomic variation in the narrow-leaf hopbush across a continental biodiversity refugium
Source: Sci Rep. 2017 Feb 1;7:41367. doi: 10.1038/srep41367 (PMC5286450; doi:10.1038/srep41367)
Supplement: Supplementary Information [file srep41367-s1.pdf]

Targeted capture to assess neutral genomic variation in the narrow-leaf hopbush across a continental biodiversity refugium

Matthew J. Christmas, Ed Biffin, Martin F. Breed and Andrew J. Lowe

Supplementary Methods

Flags used in SNP calling using mpileup:

-l selected.snps.list – use a pre-defined list of alleles for genotyping rather than selecting SNPs on basis of deviation from the reference base

-f use a fasta format sequence reference

-b bam.file.list – perform the genotyping on the list of filenames contained within this file

-I = skip INDELs

-C 50 = adjust the mapping quality to 50 – as recommended by samtools authors

-t DP,SP = include (high quality) depth-of-coverage and strand bias information in the VCF output file

-v = output results in VCF format

-A = do not discard anomalous read pairs (the reference collection is fragmented and it is seen that a fraction of ~10% of mapping reads are anomalous in the flagstat section)

-E = recalculate BAQ on the fly – the base mapping quality information is used in calculation of likelihood that a variant is real / noise
